# Imaging cellular pharmacokinetics of ^18^F-FDG and 6-NBDG uptake by inflammatory and stem cells

**DOI:** 10.1371/journal.pone.0192662

**Published:** 2018-02-20

**Authors:** Raiyan T. Zaman, Silvan Tuerkcan, Morteza Mahmoudi, Toshinobu Saito, Phillip C. Yang, Frederick T. Chin, Michael V. McConnell, Lei Xing

**Affiliations:** 1 Department of Medicine, Division of Cardiovascular Medicine, Stanford University School of Medicine, Stanford, CA, United States of America; 2 Department of Radiation Oncology, Division of Medical Physics, Stanford University School of Medicine, Stanford, CA, United States of America; 3 Department of Radiology, Molecular Imaging Program at Stanford, Stanford University School of Medicine, Stanford, CA, United States of America; University of Chicago, UNITED STATES

## Abstract

**Objectives:**

Myocardial infarction (MI) causes significant loss of cardiomyocytes, myocardial tissue damage, and impairment of myocardial function. The inability of cardiomyocytes to proliferate prevents the heart from self-regeneration. The treatment for advanced heart failure following an MI is heart transplantation despite the limited availability of the organs. Thus, stem-cell-based cardiac therapies could ultimately prevent heart failure by repairing injured myocardium that reverses cardiomyocyte loss. However, stem-cell-based therapies lack understanding of the mechanisms behind a successful therapy, including difficulty tracking stem cells to provide information on cell migration, proliferation and differentiation. In this study, we have investigated the interaction between different types of stem and inflammatory cells and cell-targeted imaging molecules, ^18^F-FDG and 6-NBDG, to identify uptake patterns and pharmacokinetics *in vitro*.

**Methods:**

Macrophages (both M1 and M2), human induced pluripotent stem cells (hiPSCs), and human amniotic mesenchymal stem cells (hAMSCs) were incubated with either ^18^F-FDG or 6-NBDG. Excess radiotracer and fluorescence were removed and a 100 μm-thin CdWO_4_ scintillator plate was placed on top of the cells for radioluminescence microscopy imaging of ^18^F-FDG uptake, while no scintillator was needed for fluorescence imaging of 6-NBDG uptake. Light produced following beta decay was imaged with a highly sensitive inverted microscope (LV200, Olympus) and an Electron Multiplying Charge-Couple Device (EM-CCD) camera. Custom-written software was developed in MATLAB for image processing.

**Results:**

The average cellular activity of ^18^F-FDG in a single cell of hAMSCs (0.670±0.028 fCi/μm^2^, P = 0.001) was 20% and 36% higher compared to uptake in hiPSCs (0.540±0.026 fCi/μm^2^, P = 0.003) and macrophages (0.430±0.023 fCi/μm^2^, P = 0.002), respectively. hAMSCs exhibited the slowest influx (0.210 min^-1^) but the fastest efflux (0.327 min^-1^) rate compared to the other tested cell lines for ^18^F-FDG. This cell line also has the highest phosphorylation but exhibited the lowest rate of de-phosphorylation. The uptake pattern for 6-NBDG was very different in these three cell lines. The average cellular activity of 6-NBDG in a single cell of macrophages (0.570±0.230 fM/μm^2^, P = 0.004) was 38% and 14% higher compared to hiPSCs (0.350±0.160 fM/μm^2^, P = 0.001) and hAMSCs (0.490±0.028 fM/μm^2^, P = 0.006), respectively. The influx (0.276 min^-1^), efflux (0.612 min^-1^), phosphorylation (0.269 min^-1^), and de-phosphorylation (0.049 min^-1^) rates were also highest for macrophages compared to the other two tested cell lines.

**Conclusion:**

hAMSCs were found to be 2–3× more sensitive to ^18^F-FDG molecule compared to hiPSCs/macrophages. However, macrophages exhibited the most sensitivity towards 6-NBDG. Based on this result, hAMSCs targeted with ^18^F-FDG could be more suitable for understanding the mechanisms behind successful therapy for treating MI patients by gathering information on cell migration, proliferation and differentiation.

## Introduction

Myocardial infarction (MI), one of the leading causes of death in the United States and worldwide, results in significant cardiomyocyte loss, myocardial tissue damage, and impairment of myocardial function [1, 2]. Cardiomyocyte loss due to MI injury is considered irreversible, with the heart lacking sufficient capacity for self-regeneration [3]. Cell-based cardiac therapies are considered an attractive therapeutic alternative to reverse cardiomyocyte loss by repairing the injured myocardium that would ultimately prevent heart failure [4]. To date, a wide variety of cell sources, both of adult and embryonic origin, have been investigated for use in heart repair, with mixed outcomes [5, 6]. Current treatments of myocardial injury only slow down the disease progression without facilitating any myocardial repair [7]. The limited mitotic capacity of cardiac cells and decreased cellularity after injury leads to suboptimal natural regenerative potential of myocardial tissue. The treatment for advanced heart failure following an MI is heart transplantation despite the limited availability of the organs. [8]. Therefore, multiple pre-clinical researches specifically employed embryonic stem cells (ESCs) to restore the heart function by regenerating myocardial tissue [9–18]. Although, these outcomes inspired numerous cell-therapy-based clinical trials, it has been difficult to obtain unequivocal evidence for robust clinical benefit [19]. Thus, there is a great need to understand the mechanisms behind a successful therapy through tracking stem cell migration, proliferation, and differentiation *in vivo* using targeted imaging molecules. In this study, we successfully investigated three different cell lines—macrophages, human induced pluripotent stem cells (hiPSCs), and human amniotic mesenchymal stem cells (hAMSCs) and their interaction pattern with two different targeted imaging molecules [^18^F]fluoro-deoxyglucose (^18^F-FDG) and 6-(N-(7-Nitrobenz-2-oxa-1,3-diazol-4-yl)amino)-6-Deoxyglucose (6-NBDG). A newly developed Radioluminescence Microscopy technique was used to gather the pharmacokinetic information [20]. This pharmacokinetic information can be useful in decision making for the specific cell selection process and corresponding imaging molecule to enable tracking therapeutic cells for their migration, proliferation and differentiation patterns in myocardial repair.

## Materials and methods

### Sample preparation

We have developed three different cell lines for this experiment to test the pharmacokinetics of two different imaging molecules─^18^F-FDG (radioluminescence) and 6-NBDG (fluorescence). All murine macrophages and human stem cells generation and their use in our experiments were conducted according to an approved protocol by the Stanford Administrative Panel on Laboratory Animal Care (IRB-31517). The procedure for culturing the different cell lines is described below.

#### Culture of murine macrophages

In this study, we used RAW 264.7 murine monocyte/macrophage cells (ab7187, Abcam, Cambridge, MA, USA). These cells were reconstituted and maintained in Dulbecco’s modified Eagle’s Medium (DMEM) containing 10% fetal bovine serum (FBS) under standard culture conditions (37°C, 5% CO_2_, humidified) until confluent.

#### Culturing of the human amniotic mesenchymal stem cells (hAMSCs)

hAMSCs were isolated from the fresh human placenta of healthy donors at the Stanford University Medical Center and placed in Hank’s Balanced Salt Solution (HBSS, Life Technologies) for transport. The amniotic membrane was carefully collected and washed several times with Dulbecco's phosphate-buffered saline (DPBS, Life Technologies). Membranes were transferred into 50 ml falcon tubes and digested in trypsin-EDTA (Life Technologies) for 1 hour at 37°C, 5% CO_2_. The digested membrane was centrifuged and the collected tissue was incubated with hAMSCs media (DMEM with 100 mg/L sodium pyruvate, 29.2 mg/ml L-glutamine in 0.85% NaCl, 20% FBS, 1% pen-strep and 10 ng/mL epidermal growth factor) with type 1 collagenase (1:1 weight to volume ratio, Life Technologies), for 2 hours at 37°C. Cells were filtered through a 100 μm and 50 μm sterile filter (BD Biosciences), respectively. The obtained cells were then centrifuged at 200 × g for 5 min and cultured in hAMSCs media. The hAMSCs were purified through their positive SSEA4 receptors using magnetic-activated cell sorting (MACS). The purified cells were then cultured.

#### Culturing of the human induced pluripotent stem cells (hiPSCs)

The hiPSCs culturing technique has been described in our previously published manuscript [21]. In brief, the monoclonal hiPSC lines were generated by infection of blood mononuclear cells (blood draws of 4ml and collected in Cell Preparation Tubes to isolate the peripheral blood mononuclear cells (PBMCs) from the whole blood by centrifugation) with non-integrating Sendai virus that delivered OCT3/4, SOX2, KLF4, L-MYC, LIN-28, short-hairpin RNA for P53 and EBNA1 in chemically defined media 2, 3. Reprogrammed PBMCs were transferred to MEF feeder cells on Matrigel-coated plates at day 3 after transfection and cultured in TeSR-E7 and sodium butyrate reprogramming medium (Stemcell Technologies). On day 20 after transfection, nascent hiPSC colonies were picked and further cultured independently on Matrigel-coated (Corning®) Petri dishes in pluripotent stem cell medium (Essential 8; Life Technologies) for 1 hour at hypoxic incubator (with 5% O_2_). The hiPSCs were used for analysis after reaching the confluency of ~75%. Pluripotency was confirmed through quantitative PCR gene expression analysis of the pluripotency genes Nanog, Oct3/4 and Sox2.

### Cell imaging protocol

The same protocol was followed for imaging macrophages, hiPSCs, and hAMSCs. 10,000 live adherent inflammatory/stem cells were seeded sparsely on Matrigel in a 0.085–0.115 mm thick 20 mm diameter glass-bottom well at the center of 35 mm dish (D35-20-1-N, In Vitro Scientific Inc.) and starved for 1 hour before incubation with either 250 μCi of ^18^F-FDG or 100 μM 6-NBDG solution (1 part 6-NBDG and 29 part of DMEM without glucose or 33 μL of 6-NBDG added to 966 μL of DMEM without glucose). After 1 hour of incubation with ^18^F-FDG or 6-NBDG, excess radiotracer or fluorescence was removed using DMEM medium without glucose (3×1 mL). After washing the cells, we acquired brightfield and radioluminescence or fluorescence micrographs. Before imaging of ^18^F-FDG induced cells, a 100 μm-thick 5 mm × 5 mm CdWO_4_ scintillator plate, a non-hygroscopic inorganic scintillator with both sides polished, was placed on top of the cells and scintillating plate was covered with 1 mL DMEM with glucose. CdWO_4_ has relatively high light yield (12,000–15,000 photon/MeV), high effective atomic number (Zeff = 64), high density (7.9 g/cm^3^), and no significant afterglow. The use of thin scintillator plates and thin bottom imaging dishes is required to accommodate the short working distance of 200 mm for the microscope objective.

### Microscopy setup

**Fig 1:**
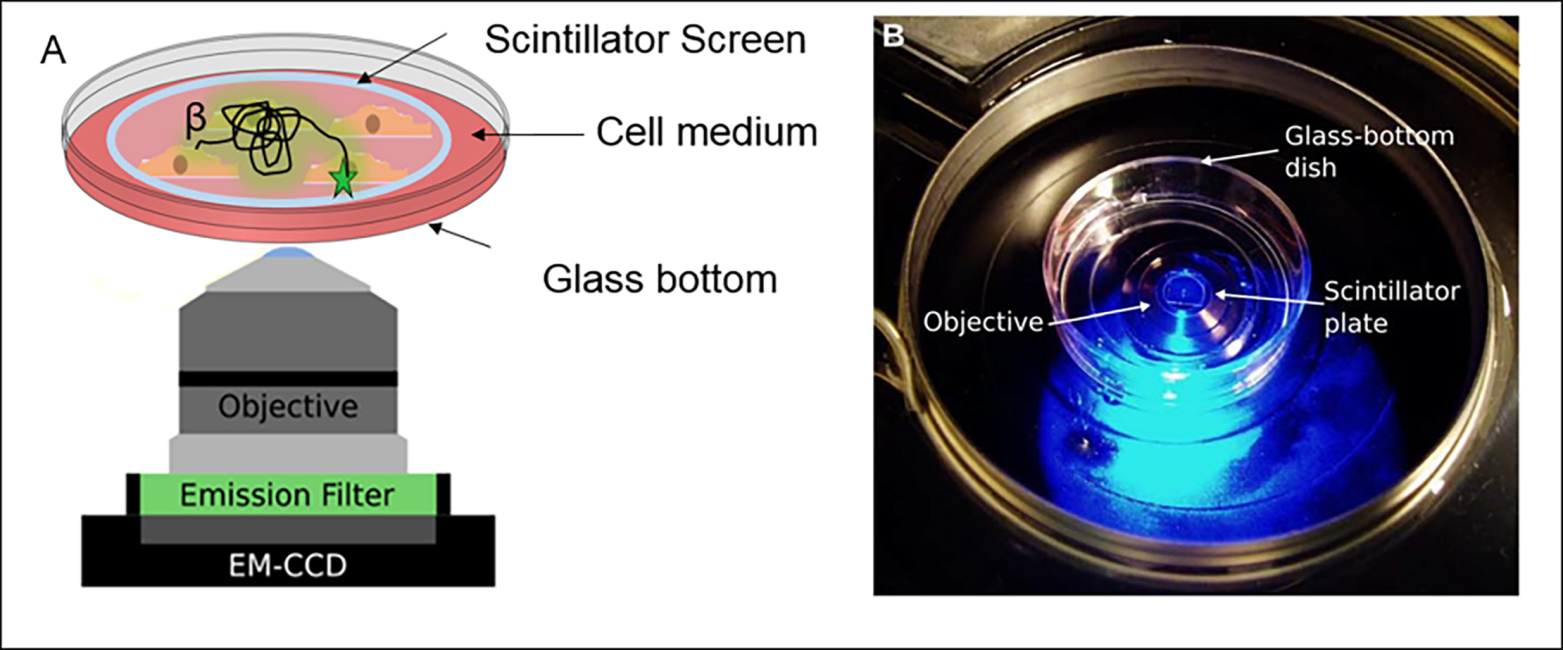
Radioluminescence microscope setup. Overview of the radioluminescence microscope. (A) Radioluminescence is produced within a scintillator plate following the emission of a beta particle from a radiotracer within a cell (green glow). The optical photons are captured by a high-numerical-aperture objective coupled to a deep-cooled EM-CCD camera. Emission and excitation filters used in combination with a light source allow for concurrent fluorescence and brightfield microscopy. (B) Photograph of the system showing a glass-bottom dish containing a scintillator plate immersed in cell culture medium and placed into the inverted microscope.

The procedure for setting up the Radioluminescence Microscope is described in our previous manuscript [20]. In short, light produced following beta decay was imaged a 300 ms exposure time with a highly sensitive inverted microscope (LV200, Olympus) fitted with a 40x/1.3 high-NA oil objective (UPLFLN40XO, Olympus), and a deep-cooled electron-multiplying charge-coupled device camera (EM-CCD, ImageEM C9100-14, Hamamatsu), cooled at -70°C (Fig 1). The C9100-14 CCD is a back-thinned frame transfer device, with a 1024×1024 array of 13 μm ×13 μm pixels. The LV200 is also equipped with temperature, humidity, and CO_2_ regulation for extended live cell imaging.

**Fig 2:**
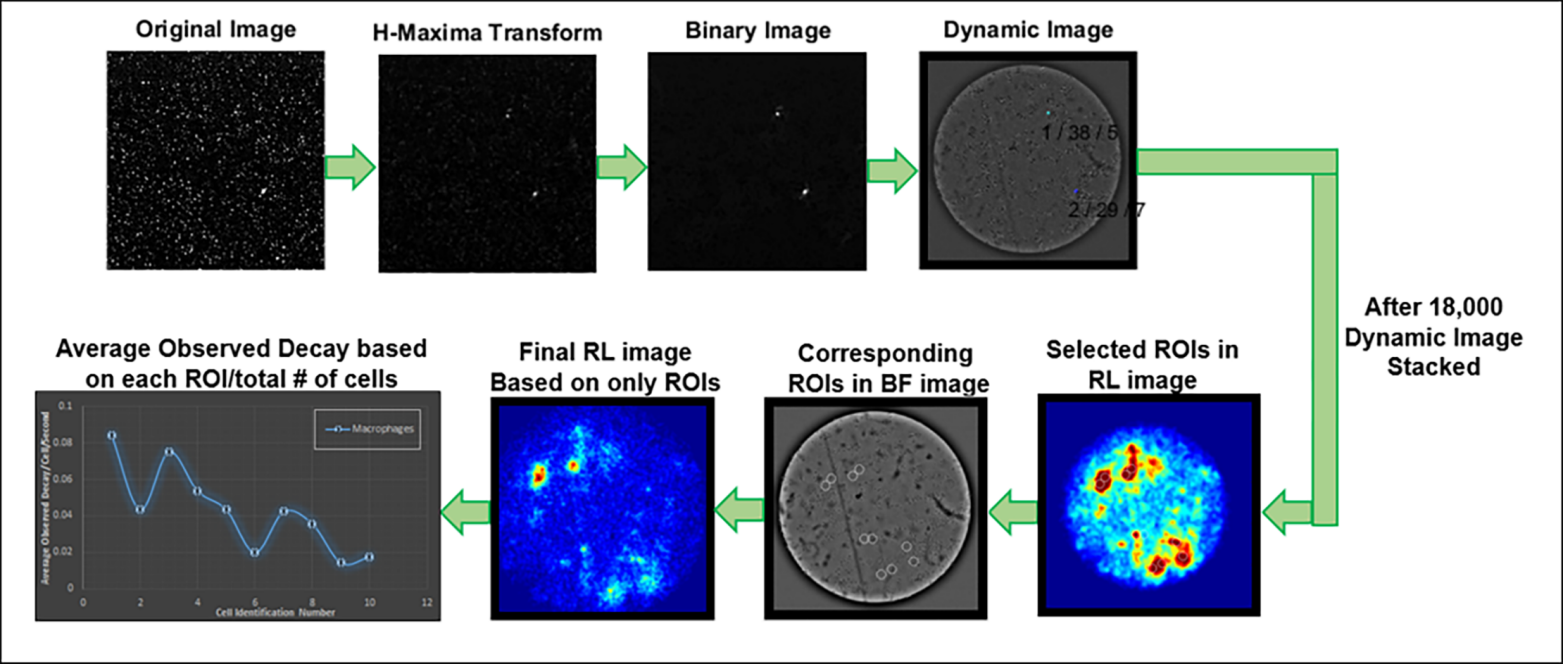
Graphical representation of the image processing protocol. Image processing for calculating average observed decay of individual macrophage cell based on radioluminescence (RL) and corresponding brightfield (BF) images. The RL image was generated from 18000 dynamic images. Each dynamic image was individually evaluated for sharpness and localization of the individual track or position. Image processing protocol described in more detail in reference [20].

**Fig 3:**
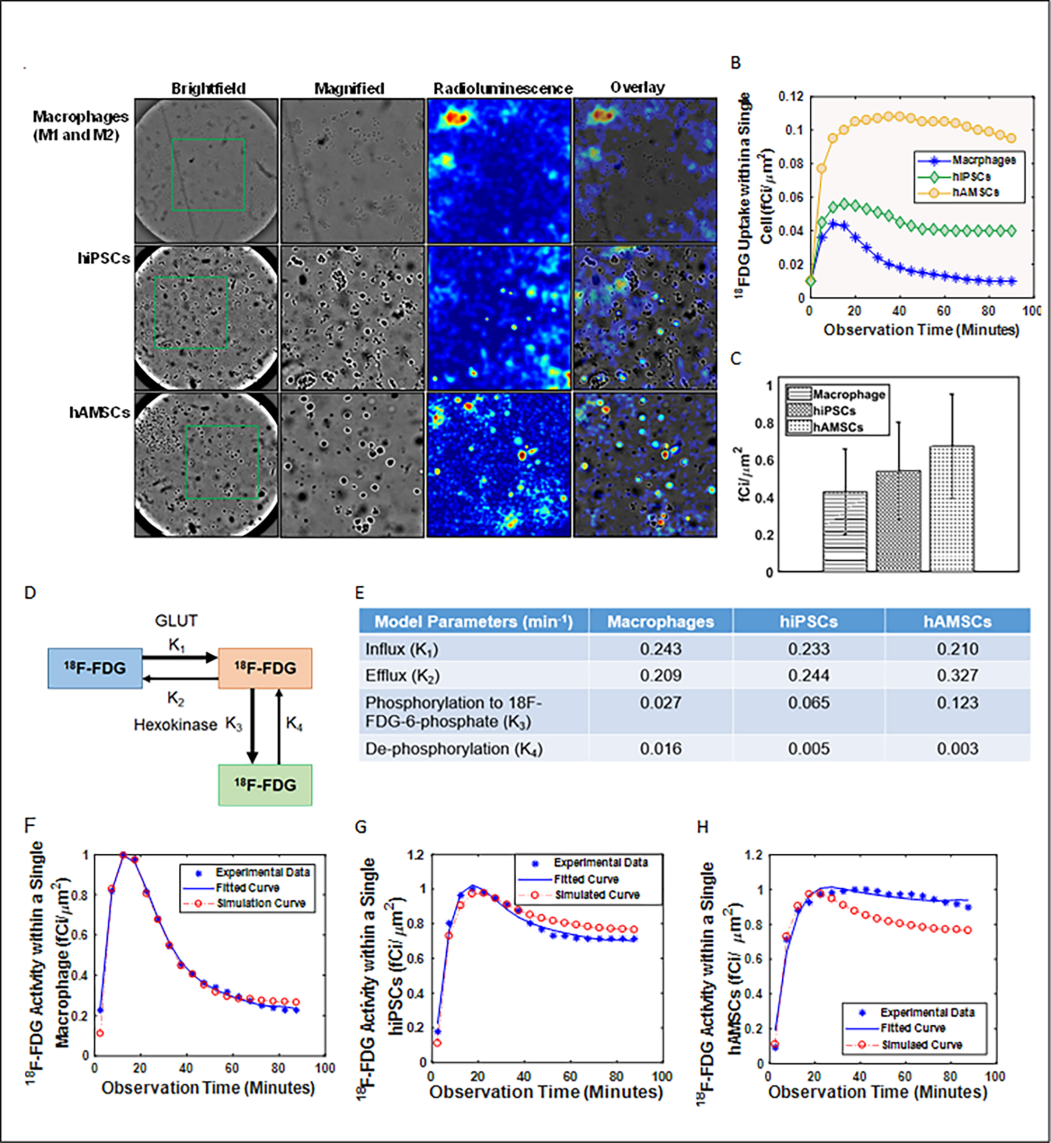
Pharmacokinetics of ^18^F-FDG uptake in a single living cell using radioluminescence microscope images. (A) Murine macrophages, human embryonic stem and mesenchymal stem cells were deprived of glucose for 1 h before incubated for 45 minutes with 250 μCi of ^18^F-FDG. (B) A dynamic representation of observed radioactivity within an individual cell over a period time. (C) Comparison between different cells and their average radiotracer uptake. (D)Two-tissue compartmental model describing FDG pharmacokinetics, including influx (*k*_1_), efflux (*k*_2_), phosphorylation to FDG-6-phosphate (*k*_3_), and dephosphorylation (*k*_4_). (E) Table listed the values of the kinetics. Compartmental analysis modeling of ^18^F-FDG pharmacokinetics consisting of normalized weighted values of *k*_1_, *k*_2_, *k*_3_, and *k*_4_ from a single cell of (F) macrophages, (G) hiPSCs, and (H) hAMSCs.

Brightfield images were acquired with no EM gain, a neutral-density filter on the excitation, and the emission shutter open. For the 40× magnification, 18,000 radioluminescence images were taken with a digital acquisition method at 10–50 ms acquisition time, an EM gain of 251/1200, 262 pixel binning, the excitation shutter closed, and the emission shutter open. Brightfield mode was used to set the microscope into focus. Optimal radioluminescence focus was achieved when the cells displayed sharp positive contrast in the corresponding brightfield image. The images were collected over 18,000 frames with 4×4 binning (1200 MHz EM Gain). Custom-written software was developed in MATLAB for image processing (Fig 2). To investigate the utility of radioluminescence microscopy for single-cell pharmacokinetic studies, we monitored the uptake of FDG in macrophages, hiPSCs, and hAMSCs over 90 minutes. We acquired serial brightfield and radioluminescence images every 5 min for 90 minutes (Fig 3A). For the 6-NBDG cell imaging the same steps were followed as above except no scintillator was added and 460 nm/535 nm filter set (Chroma Technology Corp., filter ref. D460/50x and D535/40 m) was added. In addition, two additional images were acquired—(1) background image (2) image of the petri-dish with 6-NBDG stock without cell sample.

### Image processing

The image processing procedure of radioluminescence images is described in our previous manuscript [20]. In short, image correction and analysis were performed using a custom-written software developed in MATLAB R2014a (Mathworks, Natick, MA). The correction was applied to the radioluminescence images by subtracting a dark image, taken with the same exposure time but with a non-radioactive sample in the microscope. These images were further corrected for field flatness, specific to the CCD camera used in the radioluminescence microscope, using a flat-field calibration map acquired using a uniform distribution of FDG. Gaussian filtering was also applied where necessary to reduce noise. Hot spots also get generated from long exposures due to high-energy photons (gamma rays and annihilation photons) interacted with the CCD. However, these hot spots were removed by applying a custom algorithm that can detect sharp features well above neighboring pixels.

Each radioluminescence image was corrected for radioactive decay. To measure radiotracer uptake in single cells, circular regions-of-interest (ROIs; diameter, 24 μm) were manually placed on the cells using the brightfield image. Similar ROIs were also placed in the background image as controls. Single cell radiotracer uptake was defined as the mean pixel intensity within the ROI of the corrected radioluminescence image. The same ROI analysis technique was applied for the 6-NBDG fluorescence images. For each cell type, 20 different ROIs were selected to perform statistical analysis on the average observed decay in each cell.

### Radiotracer/fluorescence molecule kinetic modeling

**Fig 4:**
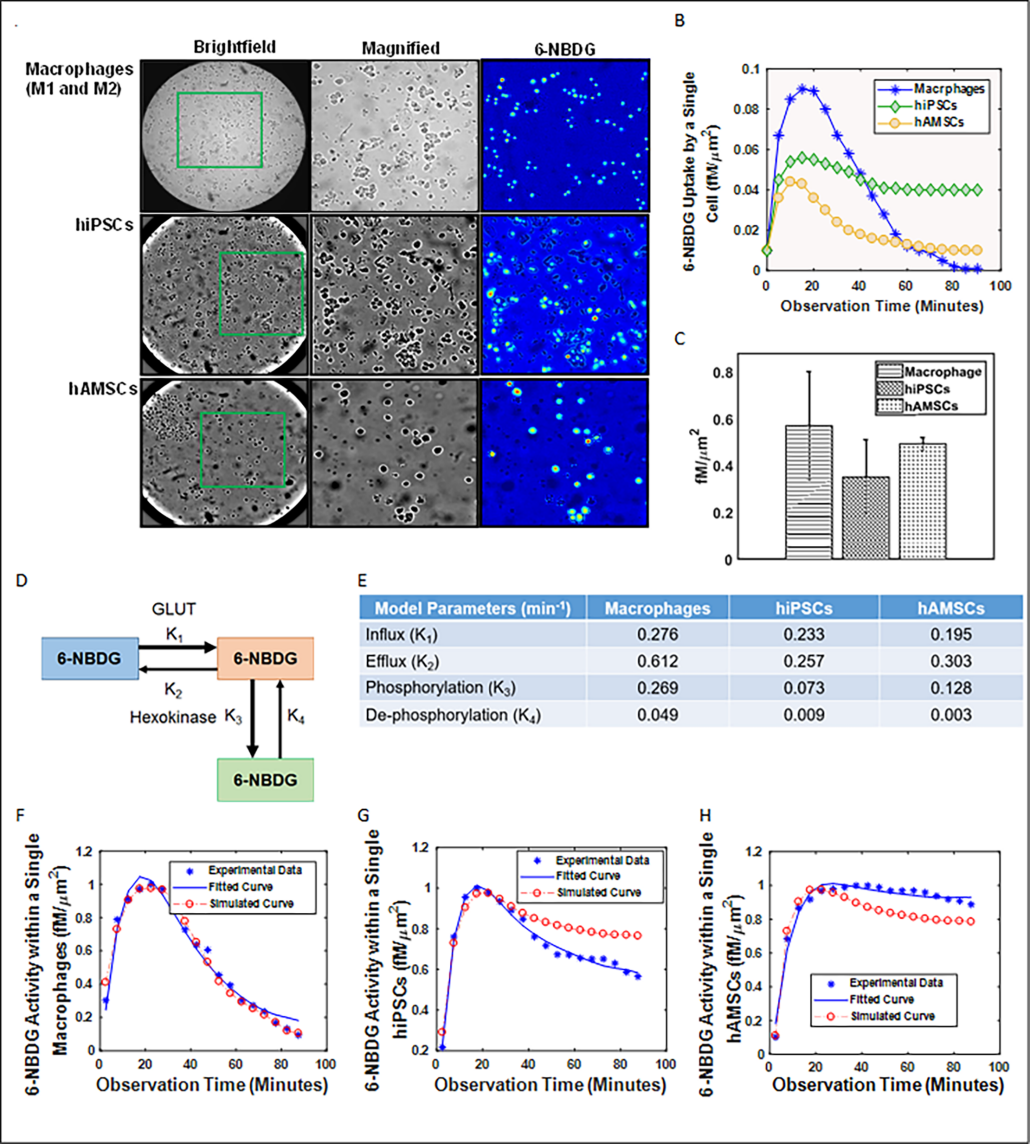
Pharmacokinetics of 6-NBDG uptake in a single living cells using fluorescence microscopy images. (A) Murine macrophages, human embryonic stem and mesenchymal stem cells were deprived of glucose for 1 h before incubated for 45 minutes with 100 μM of 6-NBDG solution. (B) A dynamic representation of observed fluorescence activity within an individual cell over a period time. (C) Comparison between different cells and their average 6-NBDG uptake (D) Two-tissue compartmental model describing 6-NBDG pharmacokinetics, including influx (*k*_1_), efflux (*k*_2_), phosphorylation to FDG-6-phosphate (*k*_3_), and dephosphorylation (*k*_4_). (E) Table listed the values of the kinetics. Compartmental analysis modeling of 6-NBDG pharmacokinetics consisting of normalized weighted values of *k*_1_, *k*_2_, *k*_3_, and *k*_4_ kinetics from a single cell of (F) macrophages, (G) hiPSCs, and (H) hAMSCs.

The kinetic modeling technique has been described in our previously published study [20]. In brief, the uptake and metabolism of ^18^F-FDG and 6-NBDG into glucose-deprived cells were mathematically modeled using the following two-tissue compartmental model (Figs 3D and 4D):

$$\frac{C(t)}{C_{a}}=\frac{k_{1}k_{2}}{{\left(k_{2}+k_{3}\right)}^{2}}(1-e^{-(k_{2}+k_{3})t})+\frac{k_{1}k_{3}}{k_{2}+k_{3}}t$$

where *C*_*a*_ is the extracellular ^18^F-FDG or 6-NBDG concentration (consider as constant); *C*(*t*) is the time-dependent intracellular FDG/NBDG concentration (including free FDG/NBDG and bound FDG-6-P, NBDG-6-P); and *k*_1_ (influx), *k*_2_ (efflux), and *k*_3_ (irreversible phosphorylation) are the rate constants of FDG/NBDG [22]. *k*_1_ of ^18^F-FDG and 6-NBDG in cells (as shown in Figs 3A and 4A) was quantified by Linear Integration analysis using COMCAT.

For $t>>\frac{1}{k_{2}+k_{3}}$, the exponential term to be considered negligible leads to the intracellular and extracellular compartments to be in equilibrium. Therefore, the intracellular concentration of ^18^F-FDG rising linearly with time due to irreversible trapping. The slope and intercept of this linear rise are the Patlak coefficients [23]. We used non-linear weighted least squares curve fitting to estimate the parameters of the model. The fitting weights were adjusted to decrease the contribution of later time points, which have higher noise due to radioactive decay.

*k*_2_ of FDG/NBDG from cells was modeled using a two-tissue compartmental model:

$$C(t)=a_{1}e^{-\lambda_{1}t}+a_{2}e^{-\lambda_{2}t}$$

Where, *a*_1_ and *a*_2_ are positive coefficients, dependent upon initial conditions; *λ*_1_ and *λ*_2_ are the eigenvalues of the differential system of equations describing transport of ^18^F-FDG/6-NBDG between compartments. The rate constant of *k*_4_ models the possible de-phosphorylation of ^18^F-FDG-6-phosphate or 6-NBDG-6-phosphate (FDG-6-P, NBDG-6-P). Although, *k*_4_ was included in this model but it assumed to be much smaller than *k*_3_. Furthermore, due to the large extracellular volume, the concentration of ^18^F-FDG/6-NBDG in the cell culture medium was assumed to remain negligible after withdrawal of ^18^F-FDG/6-NBDG (washed off after 1 hour of incubation). Under these assumptions, the eigenvalues can be approximated as

$$\lambda_{1}=k_{2}+k_{3}+\frac{k_{2}k_{4}}{k_{2}+k_{3}}$$

and

$$\lambda_{2}=\frac{k_{2}k_{4}}{k_{2}+k_{3}}$$

These rate parameters were estimated by fitting the *k*_2_ model to the measured time-activity curves. For cells for which the solution of the fit yielded *λ*_1_ ≈ *λ*_2_ or *λ*_2_ < 1 min^-1^, the *k*_2_ curve was fitted with a single exponential function. In the special case of irreversible trapping (*k*_4_ = 0), the model is described by a single exponential decay with rate *λ*_1_ = *k*_2_ + *k*_3_.

### Statistical analysis

A pairwise two-sample Student’s t-test was performed to compare radioluminescence and fluorescence signal intensity from the ^18^F-FDG and 6-NBDG-enriched macrophages, hiPSCs, and hAMSCs. The underlying distribution was found to be normally distributed per QQ-plots for all samples. As the number of cells and dose were the same for all three cell types, these factors were not considered in the statistical analysis. These samples were not randomized. In vitro analyses were performed using MATLAB software. We presented all values as mean ± standard deviation. We considered P<0.01 as statistically significant for all in vitro analyses.

## Results

We observed good co-localization between the radioluminescence and fluorescence intensity and the cell outline seen on brightfield images based on the acquired brightfield, radioluminescence and fluorescence micrographs (Figs 3A and 4A). Although there were significant variance identified in the average uptake of ^18^F-FDG and 6-NBDG for all three cell lines, the relationship between cell-to-cell comparisons was found to be linear for macrophages, hiPSCs, and hAMSCs.

The average cellular activity of ^18^F-FDG in a single cell of hAMSCs per minute (0.670±0.028 fCi/μm^2^, P = 0.001) was 20% and 36% higher compared to hiPSCs (0.540±0.026 fCi/μm^2^, P = 0.003) and macrophages (0.430±0.023 fCi/μm^2^, P = 0.002), respectively (Fig 3C). These result are statistically significant (P<0.01). The average cellular activity of 6-NBDG in a single cell of macrophages per minute (0.570±0.180 fM/μm^2^, P = 0.004) was 38% and 14% higher compared to hiPSCs (0.350±0.140 fM/μm^2^, P = 0.001) and hAMSCs (0.490±0.050 fM/μm^2^, P = 0.006), respectively (Fig 4C).

The hAMSCs exhibited the slowest influx (0.210 min^-1^) but the fastest efflux (0.327 min^-1^) rate compared to the other tested cell lines for ^18^F-FDG (Fig 3E). This cell line also has the highest phosphorylation (0.123 min^-1^) but exhibited the lowest rate of de-phosphorylation (0.003 min^-1^; P<10^−5^, r = -0.94). For ^18^F-FDG, the hiPSCs and hAMSCs exhibited higher efflux compared to influx rate unlike macrophages. All cell lines showed higher phosphorylation than dephosphorylating toward ^18^F-FDG. However, the uptake pattern for 6-NBDG was very different in these three cell lines. The influx (0.276 min^-1^), efflux (0.612 min^-1^), phosphorylation (0.269 min^-1^), and de-phosphorylation (0.049 min^-1^) rate were highest for macrophages compared to the other two tested cell lines and it showed inverse correlation between influx/efflux (P<10^−5^, r = -0.93) and phosphorylation/de-phosphorylation (P<10^−5^, r = -0.91). For 6-NBDG, all cells exhibited a faster efflux rate compared to influx rate and a higher phosphorylation compared to de-phosphorylation.

Although, ^18^F-FDG uptake varied significantly from cell to cell, all three different cell types displayed the same linear increase in radioactivity for first 20 minutes. However, after 20 minutes a faster decrease was observed for macrophages followed by a plateau at 40 minutes (Fig 3B and 3F). For both hiPSCs and hAMSCs, a slow decrease in activity was observed after 20 minutes (Fig 3B, 3G and 3H). Although, macrophages displayed similar linear increase in 6-NBDG for first 20 minutes followed by a faster decay for 60 minutes and reached a plateau after 80 minutes (Fig 4B and 4F). However, hiPSCs and hAMSCs exhibited similar pattern in 6-NBDG uptake as ^18^F-FDG (Fig 4B, 4G and 4H).

## Discussion

In this study, to the best of our knowledge, we are the first to successfully investigate radioluminescence and fluorescence microscopy for detailed imaging and pharmacokinetic modeling, at the single cell level of inflammatory and stem cells and their uptake of targeted imaging molecules ^18^F-FDG and 6-NBDG. Based on our preliminary data, hAMSCs were found to be more sensitive towards ^18^F-FDG uptake compared to hiPSCs and macrophages. However, macrophages exhibited the most sensitivity to 6-NBDG uptake.

^18^F-FDG is preferentially taken up and retained in high concentration within hAMSCs compared to the other two cell lines due to high glucose metabolism. The *k*_1_ rate of ^18^F-FDG towards hAMSCs is the slowest but it has the fastest *k*_2_ rate among the three cell lines. The *k*_1_/*k*_2_ and *k*_3_/*k*_4_ rate of all cell lines were inversely correlated. However, the uptake pattern for 6-NBDG was very different in these three cell lines. The average activity of 6-NBDG in a single cell of macrophages per minute was higher compared to hiPSCs and hAMSCs. Similar inverse correlation was also observed between influx/efflux and phosphorylation/de-phosphorylation. The efflux rate of 6-NBDG is higher than influx rate in all three cell lines. In other words, 6-NBDG accumulated slowly within the cells, but depleted faster.

Single cell time-activity curves were found consistent with the Patlak’s model for the early time point. However, equilibrium was established after a short transient period (after 20 minutes) due to linear increase of intracellular concentration of ^18^F-FDG with time and irreversible trapping of ^18^F-FDG into the cell. This finding is similar to our previously published study for breast cancer cells [20]. The slope of the linear rise is the product of *k*_1_ rate and fraction of the intracellular ^18^F-FDG irreversible metabolization. These slopes are similar for each cell lines. However, macrophages showed much faster decline in ^18^F-FDG activity compared to hiPSCs and hAMSCs after 20 minutes from the initial uptake.

We found that the majority of cells stopped accumulating ^18^F-FDG/6-NBDG within 20 minutes of the initial exposure of ^18^F-FDG or 6-NBDG followed by a slow decrease in cell ^18^F-FDG or 6-NBDG concentration in hiPSCs and hAMSCs unlike macrophages. The non-negligible rate of *k*_4_ could be the likely contributing factor to this effect due to ^18^F-FDG/6-NBDG concentration reaching a steady plateau due to equilibration of phosphorylation and dephosphorylation. The slow decrease that was observed instead may have been caused by increased competition from unlabeled 2DG (a byproduct of ^18^F-FDG/6-NBDG synthesis) as ^18^F-FDG/6-NBDG concentration diminished due to radioactive decay or photo-bleaching, respectively.

Furthermore, the radioluminescent and fluorescent signals intensity varied significantly from cell to cell, indicating heterogeneous uptake of ^18^F-FDG/6-NBDG. Furthermore, solving for the pharmacokinetic coefficients such as *k*_1_, *k*_2_ and *k*_3_ showed that *k*_1_ and *k*_2_ for ^18^F-FDG and 6-NBDG were inversely correlated similar to *k*_3_ and *k*_4_. The single-cell radioluminescent signal was correlated with fluorescent signal. An exact correlation between ^18^F-FDG and 6-NBDG is not expected due to (i) possibly distinct transport mechanisms [24]; and (ii) the inability of 6-NBDG to fluoresce after being metabolized. Similar uptake pattern was observed in a the first study done with radioluminescence microscopy using 9-(4-[18F]Fluoro-3-hydroxymethylbutyl)-guanine (FHBG, PET radiotracer for transgene expression) in cancer cells that were heterogeneously transfected to express the mutant herpes simplex virus type 1 truncated thymidine kinase [20].

A limitation of this study is that we did not investigate all possible cell lines and contrast agents. Also, longer term observation of these cells and their interactions with imaging molecules would be necessary for future study in humans. Another limitation is that there are significant intrinsic differences and variability in cellular metabolism/behavior routinely observed in vitro vs. in vivo, including cell signaling, stroma and immune interaction, and Oxygen (O_2_) tension. However, based on these promising results, in future we hope to test a wider variety of cell lines associated with precise targeting molecules. Thus, we will be able to perform in vivo stem cell tracking for regenerative therapy in MI patients through targeted imaging molecules to provide information on cell migration, proliferation and differentiation.

## Conclusions

We found hAMSCs were 2–3× more sensitive to ^18^F-FDG uptake than macrophages, while macrophages exhibited the most sensitivity towards 6-NBDG uptake. Based on this result, hAMSCs targeted with ^18^F-FDG could be more suitable for understanding the mechanisms behind successful cell therapy for treating MI patients.

## Supporting information

S1 File Decay-constant. (XLSX)
